# Mechanistic Understanding of Protein–MOF Integration Through Surfactant‐Driven Interfacial Design

**DOI:** 10.1002/advs.76011

**Published:** 2026-06-09

**Authors:** Ehsan Rashidniyaghi, Mohammad Khavani, Carlie Coerver, Ruibin Liang, Raheleh Ravanfar

**Affiliations:** ^1^ Department of Chemistry and Biochemistry Texas Tech University Lubbock Texas USA

**Keywords:** interfacial designs, metal–organic frameworks, molecular mechanisms, proteins, surfactants

## Abstract

Integration of proteins into metal–organic frameworks (Protein@MOF) offers an effective strategy for protein stabilization across materials and biomedical applications. However, the molecular mechanism of protein–MOF interactions remains poorly understood, limiting rational control over their chemical and physical properties. Here, we develop a surfactant‐guided strategy to modulate the assembly of protein@MOF through interfacial design. We discovered that the interfacial environment between proteins and MOFs is the dominant factor controlling encapsulation efficiency, structural integrity, and functional performance. Lipid‐based non‐ionic surfactants such as glycerol monooleate (GMO) increase the protein's solvent‐accessible surface area (SASA), suggesting partial remodeling of the protein surface and hydration shell. Interfacial GMO enhances protein encapsulation by 20% and accelerates MOF growth by 30%. Importantly, for horseradish peroxidase (HRP) encapsulated in MOF, incorporation of lecithin results in up to a six‐fold enhancement in retained bioactivity and a near 60‐fold increase in *k*_cat_. All‐atom molecular dynamics simulations reveal concentration‐dependent, domain‐specific interactions between the surfactant and flexible surface residues via electrostatic and hydrophobic contacts. These findings establish surfactant‐driven interfacial design as a general molecular strategy to enhance protein@MOF stability and function, enabling robust alternatives to lipid nanodiscs for membrane protein stabilization and advancing applications in biocatalysis, biosensing, and drug delivery.

## Graphical abstract

This study reveals how surfactant‐driven interfacial design governs the assembly and stability of protein@MOF composites. Using lipid‐based nonionic surfactants, we modulate protein–MOF interactions to improve encapsulation efficiency, MOF crystallization, and catalytic performance. Molecular dynamics simulations uncover residue‐specific interactions at the interface, providing mechanistic insight into the role of surfactants in guiding protein integration into porous frameworks.

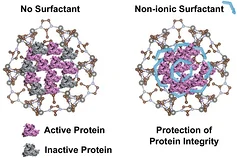

## 1 Introduction

Proteins drive essential biological processes in living systems with exceptional efficiency, selectivity, and structural adaptability [1]. These traits have motivated widespread efforts to harness proteins as functional materials in applications ranging from catalysis and sensing to targeted therapeutics [2, 3, 4, 5]. In nature, protein‐based materials, spanning from cytoskeletal filaments to silk fibers, exemplify how hierarchical structure and dynamic responsiveness arise from carefully orchestrated interactions between individual building blocks [6, 7, 8, 9, 10]. Designing synthetic analogs with similar precision, however, remains challenging, as it requires fine‐tuning of intermolecular forces that govern folding, assembly, and long‐term stability [11]. A variety of encapsulation matrices have been explored to stabilize sensitive molecules; however, among the most promising strategies to stabilize and spatially organize proteins is the use of metal–organic frameworks (MOFs), highly porous, modular architectures assembled from inorganic nodes and organic linkers [12, 13, 14, 15]. Their high surface area, tunable chemistry, and mild aqueous synthesis conditions enable biomacromolecule encapsulation with minimal structural disruption. Recent advances in MOF engineering have increasingly embraced biomimetic strategies not only for structural control but also for functional integration. For example, 1D MOF‐based silk‐like materials have been fabricated by harnessing directional MOF growth to form fibrous architectures with mechanical flexibility and hierarchical order, mimicking natural silk assembly [16]. Similarly, 2D MOF‐based films have been developed by confining MOF crystallization at interfaces, enabling the formation of thin, flexible, and highly porous sheets suitable for membrane separation and sensing applications [17]. These examples highlight the versatility of biomolecular templates and interfaces in directing MOF morphology and functionality. Particularly, 3D zeolitic imidazolate frameworks (ZIFs) offer biocompatible synthesis pathways that support room‐temperature self‐assembly, preserving the functional conformation of sensitive proteins [14, 18, 19, 20, 21]. Within this context, significant effort has been directed toward regulating protein performance inside MOFs through targeted engineering strategies [22, 23]. For instance, modification of protein surface residues to tune net charge has been shown to accelerate self‐nucleation and maintain near‐native enzymatic activity in systems such as HRP and cytochrome c [22]. Complementary approaches focus on confinement effects; for example, hollowing MOF microcrystals to generate freestanding enzyme environments can substantially enhance catalytic performance by alleviating restrictive host–guest interactions [23]. From a materials standpoint, these effects are further influenced by the intrinsic properties of the MOF scaffold. Systematic comparisons across Zn‐based ZIFs reveal that biomineralization is highly framework‐dependent, with topologies such as ZIF‐8, ZIF‐90, and ZIF‐zni identified as particularly effective for maintaining protein activity. Among these, ZIF‐8 remains the most widely employed due to its balanced pore architecture, chemical stability, and protective microenvironment [24]. In parallel, additive‐based strategies, including the use of polymers such as polyvinylpyrrolidone (PVP) and small molecules like amino acids, have been widely adopted to stabilize proteins and modulate mineralization conditions [25, 26]. More recently, advances in machine learning, particularly Bayesian optimization frameworks, have enabled rapid exploration of multidimensional experimental parameters, leading to near‐complete activity recovery for enzymes such as glucose oxidase and high retention for catalase under optimized conditions [22]. Collectively, these developments align with emerging mechanistic insights indicating that proteins do not function as rigid templates in bulk solution; rather, they primarily interact with nascent crystal surfaces, where interfacial phenomena govern nucleation and growth pathways [24, 27]. Despite this progress, the molecular mechanisms governing protein@MOF assembly at these interfaces remain poorly understood [28, 29, 30]. Central to this challenge is the interfacial environment itself, where electrostatic interactions, hydrogen bonding, and hydrophobic effects dictate nucleation, encapsulation efficiency, and protein conformation [18, 31, 32]. A critical gap therefore lies in the rational design of protein–MOF interfacial interactions, particularly in understanding how surfactants and lipid‐based amphiphiles modulate these processes.

**FIGURE 1:**
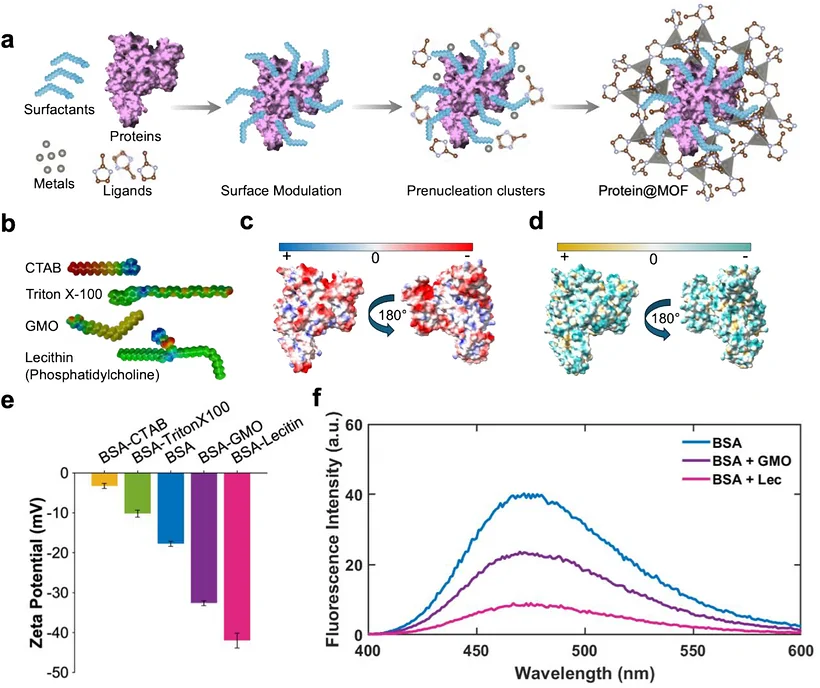
Synthesis and characterization of BSA@MOF. (a) Schematic representation of MOF growth on the BSA surface in the presence of surfactants. (b) The structure of four non‐ionic and ionic surfactants; blue: highly hydrophilic (polar) regions, green: intermediate or neutral hydrophobicity, yellow/orange/red: increasingly hydrophobic (nonpolar) regions. (c) Schematic representation of BSA's electrostatic potential, red: negative potential, white: zero, blue: positive potential, PDB ID: 4F5S. (d) Schematic representation of BSA's hydrophobicity distribution, dark cyan for most hydrophilic and dark goldenrod for most hydrophobic, PDB ID: 4F5S. (e) Comparison of the zeta potentials for native BSA and BSA treated with various surfactants. f) Fluorescence emission spectra of ANS‐bound BSA in the absence and presence of surfactants, lecithin, and GMO.

In this study, we introduce a biomimetic strategy for engineering the protein@MOF architectures through surfactant‐mediated interfacial design (Figure 1a and Figure S1). Inspired by the stabilizing role of lipid membranes in preserving protein structure and function, this approach leverages the amphiphilic and electrostatic properties of surfactants to modulate the interfacial environment of proteins during MOF formation. We anticipate that mimicking such membrane‐like flexibility at the protein–MOF boundary can improve structural retention and functional performance of encapsulated proteins. By tailoring the nature of the surfactant at the protein–MOF interface, we aim to control nucleation behavior, improve protein entrapment, and maintain structural integrity and functionality of the protein within the crystalline matrix. To implement this new concept, we selected a diverse panel of surfactants, including non‐ionic and ionic species, with varied hydrophobic tail structures and headgroup chemistries that allow interrogation of multiple interaction modes at the interface. This approach enables a systematic investigation into how surfactant properties influence protein@MOF assembly. Through combined experiments and molecular dynamics (MD) simulations, we demonstrate the feasibility of this strategy and uncover the main guiding principles for interfacial design. The key innovations in this work are twofold: (1) it significantly deepens our molecular‐level mechanistic understanding of the assembly of robust protein@MOF composites, and (2) it develops a practical and general framework for designing new bioinorganic materials with superior protein stability and catalytic efficiency, which can be applied in biocatalysis, biosensing, membrane protein stabilization, and drug‐delivery.

## 2 Results and Discussion

Within cellular environments, multivalency arising from tandem binding sites and repetitive motifs can drive the formation of biomolecular condensates [33], thereby contributing to the maintenance of intracellular crowding as well as structural and functional organization [34]. Previous work by Patterson's, Ge, and Ouyang's groups revealed that in the coprecipitation process of protein@MOF, the metal ions and organic ligands could enhance multivalent interactions with proteins [35, 36, 37, 38]. Building on this initial understanding of protein@MOF formation, we sought to investigate how the surface characteristics of the protein contribute to the assembly process. In physiological systems, cell membranes predominantly adopt lamellar liquid crystalline phases, where phospholipids self‐assemble into stable bilayers in aqueous environments [39]. However, in specialized organelles such as microsomes and mitochondria, non‐lamellar liquid crystalline structures characterized by higher curvature and dynamic flexibility are more prevalent [40]. Inspired by the critical functional roles of membrane proteins, which emerge largely through their interactions with these diverse lipid architectures [41, 42, 43], we hypothesized that introducing surfactants at the protein@MOF interface could similarly modulate interfacial properties. This adaptive interfacial environment may better accommodate the structural requirements of proteins, enhancing their compatibility with the MOF scaffold and thereby preserving both their functionality and structural integrity. To verify this hypothesis, protein@MOF was synthesized via a one‐pot coprecipitation process. Bovine serum albumin (BSA), a widely studied globular protein known for its stability and versatility, was adopted as the biomacromolecule model, and the approach was subsequently extended to horseradish peroxidase (HRP). BSA's well‐characterized surface chemistry and amphiphilic nature make it an excellent model system for studying surfactant‐mediated interfacial modulation. In a typical encapsulation process, BSA was initially dispersed in water, followed by the addition of the surfactant solution to alter the protein's interfacial properties. Subsequently, zinc nitrate and 2‐methylimidazole (HmIM) were introduced to initiate the biomimetic mineralization of ZIF‐8 around BSA, resulting in a pH increase and subsequent formation of BSA@MOF (Figure 1a,b and Table S1). In this process, the interfacial interactions between the protein and MOF precursors, including electrostatic forces, hydrophobic/hydrophilic interactions, and potential enzyme reorientation, play key roles in directing nucleation and crystallization pathways [44, 45, 46].

To systematically investigate how surfactants modulate BSA surface properties, we tested different concentrations of the cationic surfactant, cetyltrimethylammonium bromide (CTAB), along with three non‐ionic surfactants, including glycerol monooleate (GMO), lecithin, and Triton X‐100 (Figure 1b) (see Supplementary Methods for details). These surfactants are hypothesized to modulate the surface charge and hydrophobicity index of BSA (Figure 1c,d), thereby impacting the nucleation and growth behavior of BSA@MOF composites, as well as their resulting structural stability [47, 48]. The zeta potential (ζ) of BSA was measured in the presence of each surfactant to quantify the effects on protein surface charge and potential interfacial restructuring (Figure 1e).

Our findings revealed that the addition of surfactants significantly altered the zeta potential of BSA, indicating substantial modulation of interfacial charge interactions (Figure 1e). At pH 7, native BSA, with an isoelectric point (pI) of 4.9, exhibited a ζ potential of −17.8 ± 1.3 mV (Figure 1e), consistent with its anionic nature due to the deprotonation of carboxylic acid groups (glutamate and aspartate) [49]. The addition of non‐ionic lipid‐based surfactants, such as GMO and soy lecithin, led to a significant increase in negative charge density, lowering the ζ potential to −32.6 ± 1.2 mV and −42.0 ± 3.7 mV, respectively (Figure 1e). This suggests that GMO and lecithin contribute to BSA stabilization by increasing electrostatic repulsion at the protein interface, likely facilitated by hydrogen bonding and non‐covalent interactions, which help maintain structural integrity and prevent aggregation [40, 50]. GMO is a glycerol fatty acid ester characterized by a *cis* double bond at the C9 position, comprising a hydrophilic glycerol head group capable of hydrogen bonding in aqueous environments and a hydrophobic acyl tail, rendering it highly amphiphilic (Figure 1b) [40, 51]. In our formulation process, GMO was initially dissolved in ethanol and subsequently incorporated into the aqueous BSA solution. Given that GMO readily forms a cubic liquid crystalline phase (specifically, the diamond D phase) in environments containing greater than 40% water [40], it is plausible that such non‐lamellar structural organization at the protein interface contributed to the observed stabilization due to the local restructuring of the BSA hydration layer. The presence of these non‐lamellar phases may provide a dynamic, flexible interfacial environment that better accommodates the BSA molecules.

Similarly, lecithin, a natural mixture of polar lipids (glycolipids, phospholipids) and neutral lipids (triglycerides), may have facilitated a local restructuring of the BSA hydration layer due to its key role in the self‐assembly of lyotropic liquid crystalline phases [52], contributing to the pronounced decrease in zeta potential (Figure 1b,e). In contrast, Triton X‐100, another non‐ionic surfactant, and CTAB, a cationic surfactant, did not promote non‐lamellar phase transitions but instead reduced the magnitude of BSA's surface charge. Triton X‐100 caused a moderate increase in zeta potential to −10.2 ± 1.7 mV, while CTAB induced the most significant shift, raising it to −3.3 ± 1.3 mV (Figure 1e). As a bulky ethoxylated surfactant, Triton X‐100 likely intercalated its hydrophobic tail into exposed nonpolar regions of BSA, disrupting electrostatic interactions without forming an organized interfacial phase [53]. Meanwhile, CTAB effectively neutralized BSA's negative surface charge through strong electrostatic attraction between its positively charged ammonium groups and BSA's anionic residues, leading to a significant charge compensation effect [54, 55].

We also performed an experiment using 8‐anilino‐1‐naphthalenesulfonic acid (ANS) as a fluorescence probe to directly investigate changes in protein hydrophobicity upon the addition of surfactants (Figure 1f), complementing the zeta potential data. ANS is a well‐established extrinsic fluorescent probe that exhibits enhanced fluorescence intensity and blue‐shifted emission upon binding to exposed hydrophobic regions of proteins [56]. Our results demonstrate that both GMO and lecithin induce a decrease in ANS fluorescence intensity in BSA, indicating reduced accessibility of surface‐exposed hydrophobic binding sites (Figure 1f). This trend suggests that both amphiphilic lipids interact with BSA and compete with ANS for hydrophobic regions; however, the magnitude of the effect differs between the two surfactants. Lecithin produces a substantially stronger reduction in ANS fluorescence compared to GMO, which can be attributed to its distinct molecular architecture and interfacial binding capability (Figure 1f). This suggests the formation of a more continuous and tightly packed protein–lecithin interfacial layer around BSA. In the case of BSA‐lecithin specifically, the stronger suppression of ANS fluorescence reflects its ability to act as an efficient competitive and masking agent for BSA's intrinsic hydrophobic pockets. BSA contains multiple high‐affinity fatty‐acid binding sites that naturally accommodate amphiphilic ligands, making it particularly susceptible to lecithin association [57]. Lecithin can bind to these regions and/or organize into surface‐associated lipid clusters, which reduce the number of accessible hydrophobic cavities available for ANS binding.

**FIGURE 2:**
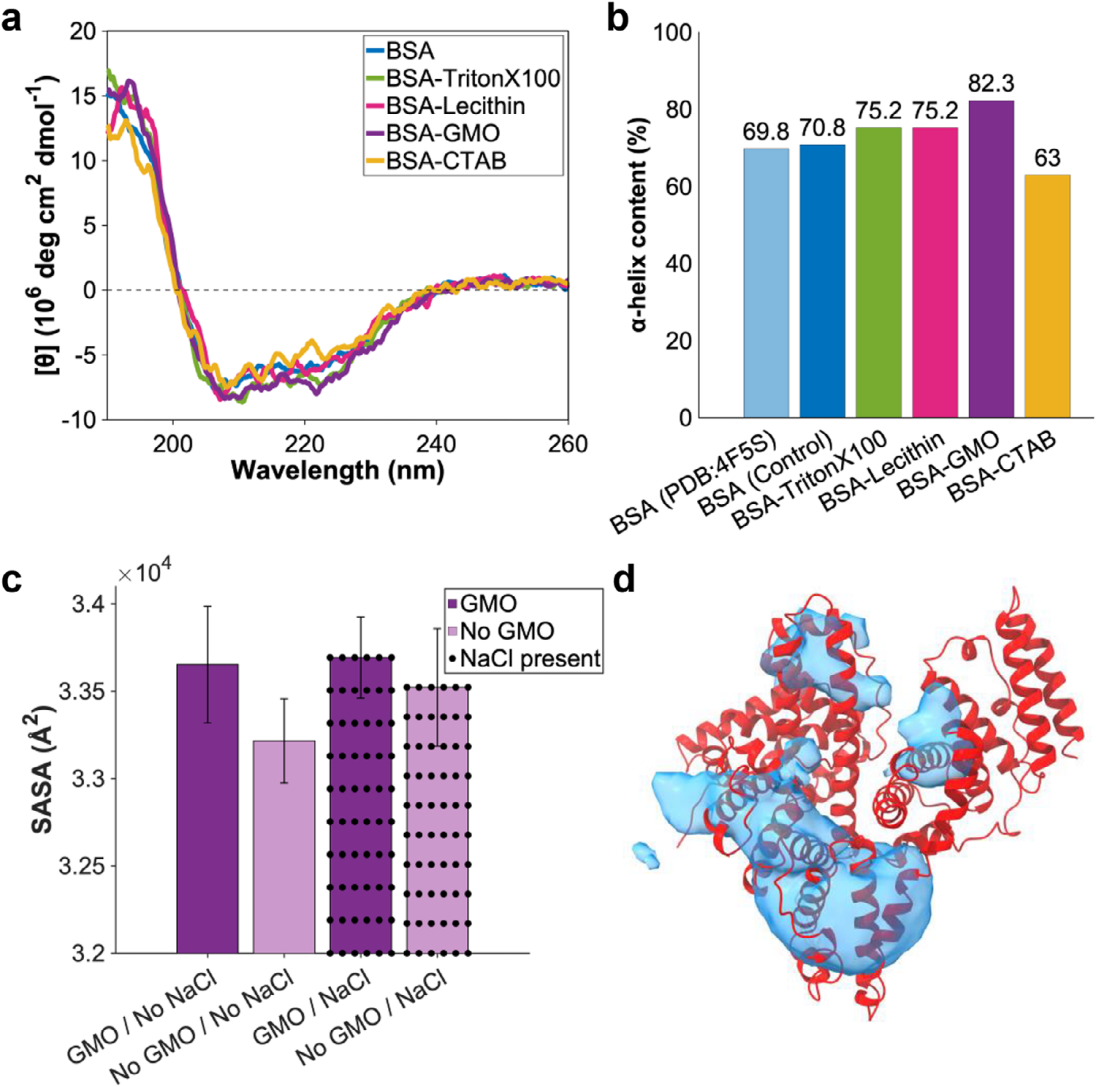
(a) Circular dichroism (CD) spectra of native BSA compared to BSA treated with various surfactants. (b) Percentage of α‐helix content in native BSA and BSA treated with surfactants. (c) Solvent‐accessible surface area (SASA) values for BSA in the presence and absence of GMO averaged over six independent MD simulations with and without NaCl concentration. (d) Spatial distribution of the surfactant head groups around BSA, represented as an isosurface with an average occupancy of 0.005. The protein is displayed in red, and the blue isosurface highlights regions around the protein visited most frequently by the head groups of GMO molecules. The occupancy isosurface was generated by averaging the center‐of‐mass positions of the GMOs’ head groups across six independent MD trajectories without NaCl concentration.

To further investigate the structural stability of BSA in the presence of surfactants, we employed far‐UV circular dichroism (CD) spectroscopy, which is sensitive to the conformational state of the protein backbone [58]. CD spectra revealed that BSA retains a predominantly α‐helical structure under all tested conditions, as evidenced by the characteristic negative ellipticity bands at 208 and 222 nm and a positive band near 193 nm (Figure 2a), consistent with its native conformation [59, 60, 61]. Quantitative secondary structure analysis using the BeStSel algorithm indicated that native BSA exhibits approximately 70% α‐helical content, in agreement with its previously reported crystal structure, PDB ID: 4F5S (Figure S2) [62]. Upon addition of surfactants, distinct trends emerged in BSA's secondary structure content. GMO led to the most pronounced increase in α‐helicity, raising it to 82%, followed by lecithin and Triton X‐100, both of which enhanced helical content to 75% (Figure 2b). This trend closely mirrors the zeta potential results, where GMO and lecithin significantly increased BSA's negative surface charge (Figure 1e). The enhancement in α‐helical structure in the presence of GMO is likely attributed to its ability to modulate the protein surface by providing a hydrogen‐bond‐rich interfacial environment that supports native protein folding [40]. Lecithin, which also promotes lyotropic liquid crystalline phase formation [52], appears to exert a similar but slightly less pronounced stabilizing effect. Triton X‐100, although non‐ionic, did not promote interfacial ordering due to its lack of a lipid‐like double‐chain structure, resulting in weaker and more transient interactions with proteins [53, 63]. In contrast, CTAB, a cationic surfactant, decreased α‐helical content to 63%, suggesting partial unfolding or conformational destabilization (Figure 2b). This loss of structure correlates with CTAB's significant neutralization of BSA's surface charge and highlights the disruptive effect of strong electrostatic binding on protein folding [54, 55].

Motivated by these findings, we sought to examine the molecular interactions at the BSA–surfactant interface using all‐atom molecular dynamics (MD) simulations in the presence and absence of the GMO molecules and a 0.15 M NaCl concentration, with particular focus on GMO's stabilizing effect on protein structure. These simulations enabled us to identify the protein residues that interact most strongly with surfactant molecules and to evaluate how these interactions are modulated by ionic strength. Moreover, solvent‐accessible surface area (SASA) values and occupancy maps were calculated (Figure 2c,d). The presence of 0.15 M NaCl was not intended to exactly match the experimental system, but rather to examine how ionic strength and electrostatic screening influence the observed interaction patterns. Since surfactant‐protein interactions can involve both hydrophobic and electrostatic contributions, introducing salt helps determine whether the identified binding regions are stable or sensitive to changes in the surrounding environment. We found that the main interaction sites remain largely consistent in both the absence and presence of NaCl (Figure 2c).

To identify preferential binding sites, we calculated the spatial distribution of GMO headgroups around the protein, which is represented as isosurfaces with occupancy beyond 0.005 (Figure 2d). Additionally, we calculated the conformational factor (*P_i_*, with *i* being the residue number) For each residue, defined as the relative contact frequency between GMO headgroups and individual amino acids across all trajectories. Residues with *P_i_* ≫ 1 were considered to exhibit above‐average interaction with GMO. Both hydrophobic and hydrophilic residues showed significant interactions with the surfactant (Figures S3 and S4), indicating that GMO binding is not limited to a single residue type. We identified residues with *P_i_* ≫ 1 that consistently appeared across simulation setups under both 0 M and 0.15 M NaCl conditions, including Glu186, Lys187, Phe205, Phe227, Thr231, Asp323, Ala324, Lys350, Arg435, Lys439, and Tyr451 (Table S1). These residues encompass a diverse set of physicochemical classes, acidic (Glu, Asp), basic (Lys, Arg), polar (Thr, Tyr), aromatic (Phe), and aliphatic (Ala). They are predominantly hydrophilic residues, highlighting GMO's ability to engage in both electrostatic and hydrophobic interactions. This molecular versatility is consistent with earlier experimental data that demonstrated GMO enhanced the magnitude of BSA's negative zeta potential (Figure 1e) and increased α‐helicity (Figure 2b), potentially through hydrogen bonding with polar residues and electrostatic interactions with charged side chains. Moreover, interactions with hydrophobic and aromatic residues (e.g., Phe227, Tyr451) suggest that GMO's acyl tail can embed into local nonpolar pockets on the protein surface, stabilizing its conformation (Table S2 and Figure 2c,d). The presence of 0.15 M NaCl reduced the *P_i_* values of several high‐affinity residues, indicating that ionic strength could modulate GMO‐BSA interactions by screening electrostatic forces (Table S2). This trend was also reflected in a reduced change in SASA in the salt‐containing system (Figure 2c), further supporting the role of ionic strength in reducing surfactant‐protein association.

**FIGURE 3:**
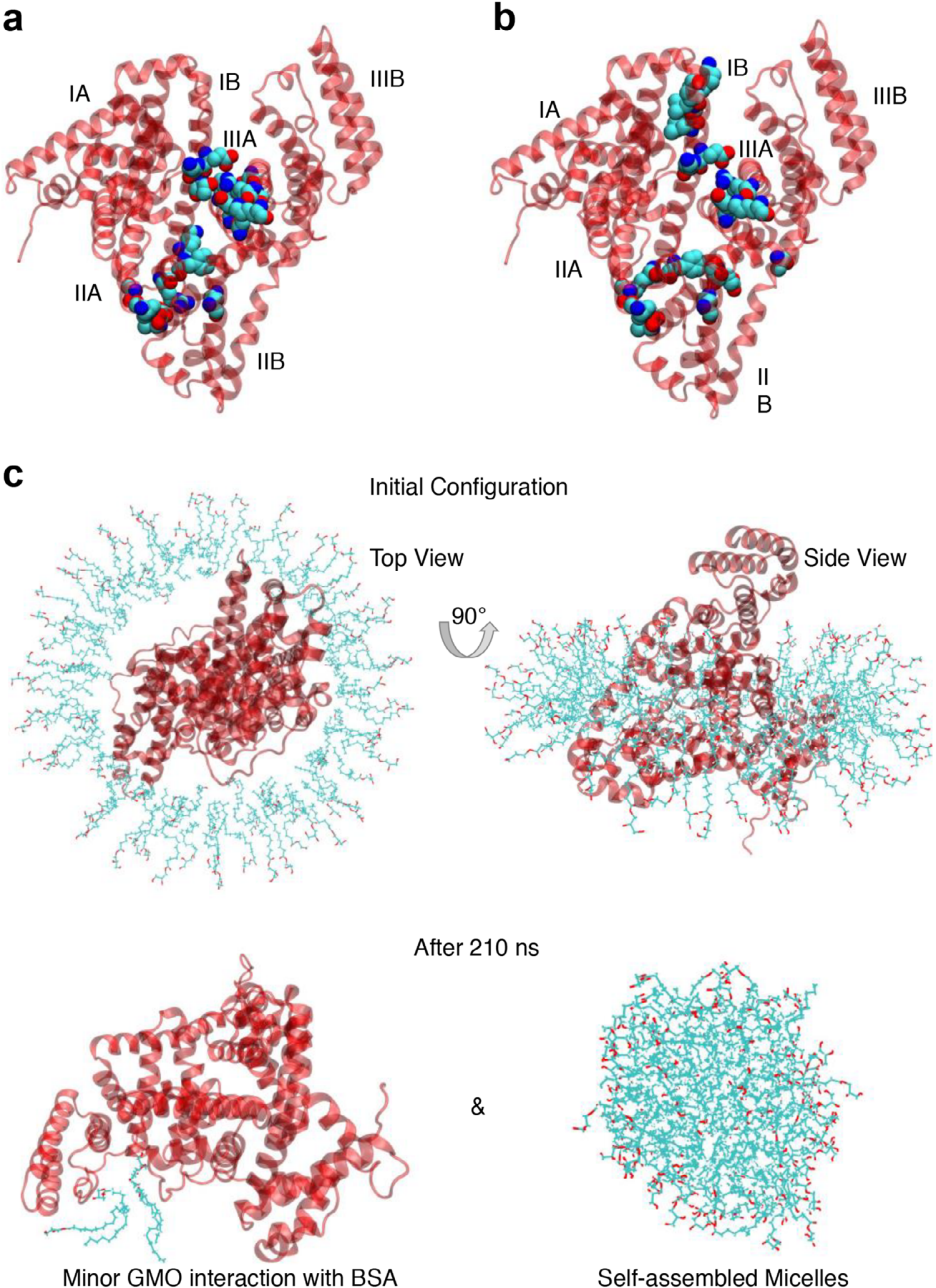
(a) BSA residues with *P_i_* > 1 with 0 M NaCl concentration. (b) BSA residues with *P_i_* > 1 in the presence of 0.15 M NaCl. The residues Glu186, Lys187, Phe205, Phe227, Tyr231, Asp323, Ala324, Lys350, Arg435, Lys439, and Tyr451 consistently exhibited above‐average interactions with GMO across all simulation setups, both with and without NaCl. These residues represent the top 20 *P_i_* values, averaged across all six independent simulations. All *P_i_* values exceed 5. The BSA domains are labeled. (c) Initial configuration of BSA and 100 GMO molecules at 0 ns, shown from top and side views, and final structure after 210 ns of MD simulation. At this higher concentration, GMO molecules exhibit reduced interaction with BSA.

The residues with highest *P_i_* values clustered within domains IIA and IIIA of BSA (Figure 3a,b), regions known to contain flexible loops and hydrophobic cavities that facilitate ligand binding [64]. This observation suggests that GMO does not bind randomly but instead localizes near functionally relevant surface regions. These regions are characterized by favorable local polarity, side chain flexibility, and surface accessibility, which together promote stable interactions. The spatial distribution of GMO headgroups supports this interpretation and clarifies how GMOs and similar surfactants can influence protein stability and solubility through targeted interactions with specific surface regions. The occupancy isosurface reveals regions where GMO headgroups have high occupancy. These regions were around domains IIA and IIIA (Figure 2d) and closely correspond to the residue‐level interaction patterns identified by the *P_i_* analysis. To assess whether GMO binding altered BSA's solvent exposure, we compared the SASA of BSA with and without surfactants under both ionic conditions (Figure 2c). The presence of GMO increased SASA relative to the control, indicating partial remodeling of the protein surface and modest conformational rearrangements that potentially perturb protein‐surfactant interactions and interfacial hydration (Figure 2c). In contrast, the inclusion of 0.15 M NaCl attenuated this effect, leading to a smaller SASA increase and a reduction in overall GMO‐protein interaction (Figure 2c).

Consistent with our above‐mentioned analysis, this indicates that the salt concentration also plays an important role in modulating the interaction between the GMO and the BSA. Moreover, the MD simulation results indicate that at higher GMO concentrations, GMO interacts less effectively with BSA (Figure 3c). In the system containing 100 GMO molecules, the surfactants aggregate to form a complete micelle, resulting in predominant GMO‐GMO interactions rather than interactions with BSA (Figure 3c). This self‐assembly behavior is potentially driven by the inherent amphiphilic property of GMO molecules, which cluster their hydrophobic tails in the inner core of the micelle and expose their headgroups to the aqueous environment. Consequently, fewer free GMO molecules are available to interact with the protein surface. These simulations were not meant to match the exact experimental conditions, but rather to understand how increasing the number of surfactant molecules around the protein changes their behavior. Our result suggests that there is an optimal range of surfactant concentrations for effective interaction with the protein. At moderate concentrations, GMO can better cover and interact with the protein surface, which may help stabilize the interface during MOF formation.

It is important to note that the MD simulations presented here do not explicitly include MOF precursors such as Zn^2+^ ions and 2‐methylimidazole. The purpose of the simulations is to isolate the interaction between the protein and surfactant molecules prior to MOF formation. Incorporating MOF precursors in a realistic way would require modeling metal‐ligand coordination, bond formation, and nucleation processes, which are not well described within standard classical force fields. Due to these limitations, we focused on a simplified system that captures the key protein–surfactant interactions in solution. These interactions are expected to play a critical role in determining how the protein surface is modified before encountering MOF building blocks during encapsulation.

Furthermore, to assess the stability of the simulation systems, we calculated the root mean square deviation (RMSD) of the protein backbone during the simulation time (Figure S5). In all cases, the RMSD increases during the initial equilibration period and then reaches a stable plateau, indicating that the systems are well‐equilibrated and do not undergo significant structural drift. In addition, the root mean square fluctuation (RMSF) analysis shows consistent residue‐level flexibility across independent simulations, with higher fluctuations primarily observed in terminal regions, as expected.

These findings are particularly important for the design of protein@MOF composites, where interfacial stabilization and controlled nucleation are critical for structural retention and encapsulation efficiency. Ge and co‐workers discovered that during the biomineralization process of protein@MOF, protein molecules form clusters with MOF precursors, contributing to structural and functional integrity and overall activity retention by sacrificing protein molecules at the cluster surface [37]. We speculate that lipid‐based non‐ionic surfactants such as GMO may reduce the extent of chemical destruction by local MOF precursors, forming a protective layer at the protein‐MOF interface while wrapping proteins inside. Previous studies have also highlighted the role of negatively charged carboxylate groups in amino acid residues in facilitating MOF nucleation and accelerating framework growth around encapsulated biomolecules [14, 18, 65]. Additionally, hydrophilicity and hydrophobicity at the protein‐MOF interface have been shown to significantly influence enzyme activity and stability upon encapsulation. For instance, hydrophilic MOFs such as ZIF‐90 and MAF‐7 effectively preserve enzymatic function by protecting proteins from denaturing conditions, whereas encapsulation in hydrophobic MOFs like ZIF‐8 often results in enzyme inactivation or minimal activity [31]. This suggests that hydrophilic enzymes favor hydrophilic MOFs, leading to better stabilization and retention of catalytic activity. However, contradictory findings indicate that hydrophobic interactions can also enhance enzyme stability. For example, MD simulations of cutinase‐encapsulated MOF‐74 (IRMOF‐74‐VI) demonstrated that hydrophobic amino acid residues (e.g., Arg) formed hydrogen bonds and salt bridges with hydrophobic linkers, maintaining structural integrity even at elevated temperatures (500 K) [32].

**FIGURE 4:**
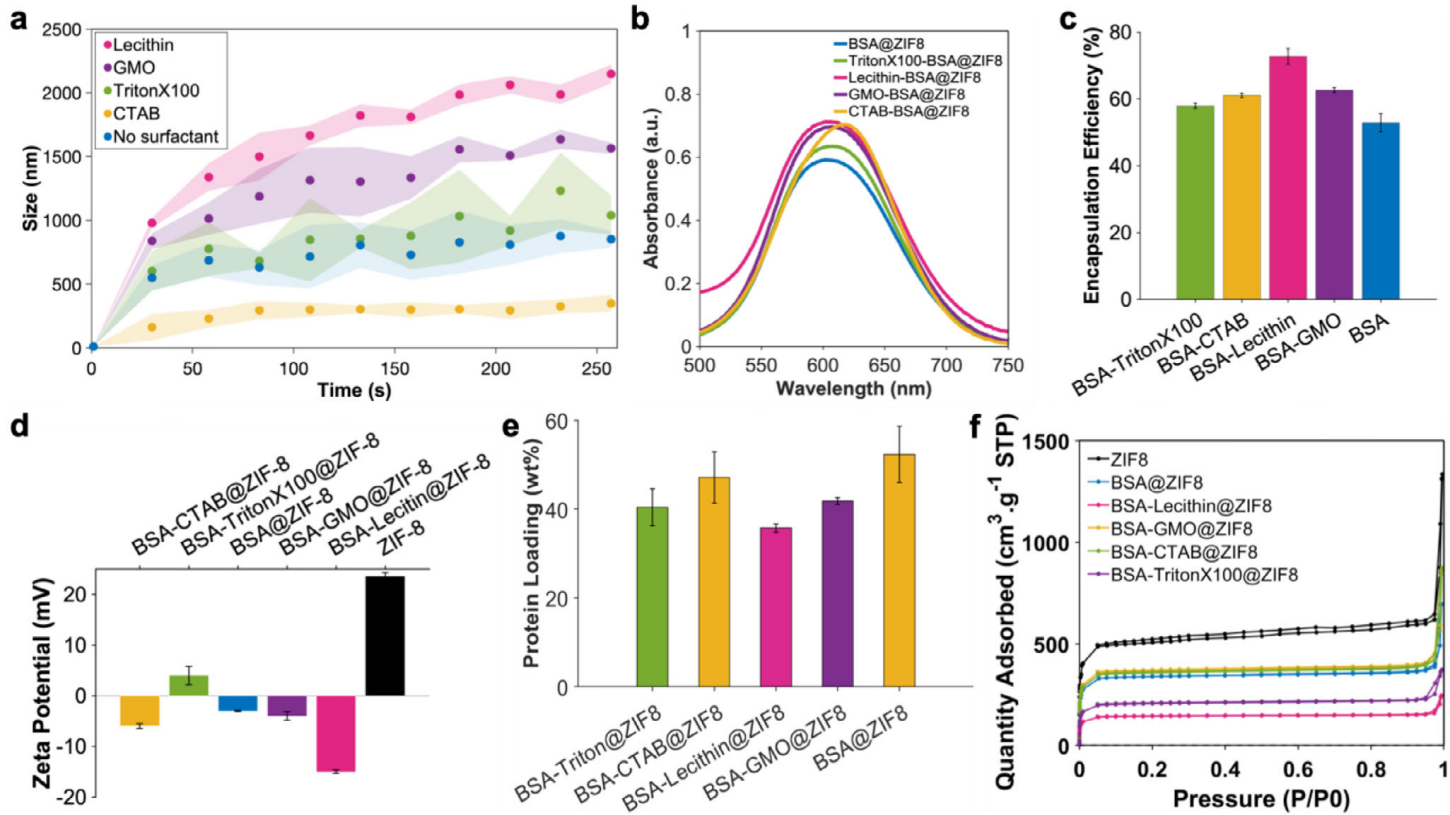
(a) Scatter plots showing the size evolution of BSA@MOF composites over 250 s, with and without surfactants. (b) Bradford assay measuring protein content in protein@MOF composites formed in the presence of different surfactants (λ_max_ = 595 nm). (c) Encapsulation efficiency (%) of BSA within MOFs synthesized with various surfactants. (d) Zeta potential of BSA@MOF composites following synthesis. (e) Protein loading (wt.%) of BSA in BSA@MOF in the presence of surfactants. (f) Nitrogen adsorption and desorption isotherms at 77 K for ZIF‐8 in comparison with the BSA@ZIF‐8 in the presence and absence of various surfactants.

To evaluate the functional impact of surfactants on biomineralization, we next examined how surfactants at the interface of proteins modulate the growth kinetics of ZIF‐8 on the BSA surface (Figure 4a, Figures S6, S7, and Table S3). Using time‐resolved dynamic light scattering (DLS), we monitored the evolution of particle size and fitted the resulting curves to an exponential model to extract initial growth rates (Figures S6, S7, and Table S3). Among the surfactants tested, lecithin resulted in the most pronounced increase in MOF growth rate, reaching 35.50 ± 4.84 nm/s. GMO followed closely with a rate of 34.30 ± 5.99 nm/s. Triton X‐100 produced a moderate growth rate of 23.28 ± 8.31 nm/s, which was not significantly different from the BSA control (27.05 ± 4.61 nm/s). Notably, CTAB markedly inhibited MOF formation, reducing the initial growth rate to 10.20 ± 3.20 nm/s (Figure 4a, Figures S6, S7, and Table S3). These results are consistent with the trend observed in zeta potential measurements (Figure 1e), where lecithin and GMO induced the largest increases in negative surface charge, potentially enhancing electrostatic interactions with zinc ions during ZIF‐8 nucleation. In contrast, CTAB significantly neutralized the negative charge on BSA, thereby suppressing nucleation and growth, consistent with its destabilizing effect on BSA secondary structure (Figure 2a). Control experiments were performed to assess the effect of surfactants on ZIF‐8 crystallization in the absence of proteins, showing no interference between the surfactants and the ZIF‐8 nucleation/growth processes (Figure S8). In parallel, we assessed the efficiency of BSA encapsulation within ZIF‐8 using a Bradford protein assay (Figure 4b,c and Figure S9). Surprisingly, the BSA control without any surfactant exhibited the lowest encapsulation efficiency across all tested conditions. In contrast, the addition of lecithin or GMO increased protein encapsulation by approximately 15%–20%, while Triton X‐100 and CTAB also produced moderate improvements (Figure 4c).

The reduced encapsulation in the control condition may reflect limited interaction between native BSA and MOF precursors due to suboptimal surface presentation or repulsive electrostatic effects [31, 32]. Notably, the CD spectra of BSA in the presence of MOF precursors prior to ZIF‐8 formation showed no significant conformational disruption relative to the BSA control (Figure S10), suggesting that changes in encapsulation efficiency are not due to unfolding but rather to interfacial compatibility. Additionally, elemental analysis revealed a clear increase in both carbon and sulfur content in the BSA@MOF samples compared to the control MOF, further supporting successful encapsulation of the protein (Figure S11). The presence of sulfur, absent in pure ZIF‐8, is attributed to sulfur‐containing amino acids such as cysteine and methionine, providing direct evidence of protein incorporation within the MOF framework (Table S4 and Figure S11) [37]. These findings further support the notion that amphiphilic surfactants, particularly lecithin and GMO, not only enhance MOF nucleation but also improve protein entrapment through modulation of interfacial structure and electrostatics.

Our results on CHNS elemental analysis reveal that all surfactant‐containing formulations achieve substantial protein loading, ranging from 36 to 52 wt.% (Figure 4e). These values are notably high relative to the recent studies [66, 67]. The work by Nikita G. and co‐workers [66] demonstrated that even at ∼96% encapsulation efficiency, protein loading reached only ∼26 wt.%, decreasing further to ∼22 wt.% at 75% encapsulation efficiency. In contrast, our system maintains significantly higher protein loadings while simultaneously achieving high encapsulation efficiencies, highlighting the effectiveness of our formulation strategy (Figure 4c,e). This behavior arises because surfactants influence both protein capture during nucleation and growth (affecting encapsulation efficiency) and the relative rate and extent of ZIF‐8 formation (affecting the final composite mass and thus loading). For instance, lecithin yields the highest encapsulation efficiency, indicating highly effective protein capture, while exhibiting moderately lower loading, consistent with increased framework formation that dilutes the protein fraction (Figure 4c,e). Conversely, BSA@ZIF‐8 prepared without surfactant shows lower encapsulation efficiency but the highest loading, reflecting reduced protein capture but a higher protein loading in the final BSA@MOF composite (Figure 4c,e). This reverse correlation between protein loading and encapsulation efficiency was also seen by previous study [66]. These results demonstrate that our surfactant‐mediated approach enables a favorable balance between efficient protein capture and high protein content in the final composite.

To investigate the impact of surfactants on the surface properties of the resulting BSA@MOF, we measured the zeta potential of the particles after MOF formation (Figure 4d). Control ZIF‐8 exhibited a highly positive zeta potential (+23.6 ± 1.5 mV), as expected due to its zinc‐rich framework. Interestingly, the presence of BSA and surfactants during the encapsulation process decreased the surface charge. Lecithin produced BSA@MOF particles with the most negative zeta potential of −15.0 ± 0.7 mV, suggesting strong interfacial interactions and effective surface modification (Figure 4d). The zeta potential results correlate well with particle morphology observed via scanning electron microscopy (SEM) (Figures S12 and S13). The particle size was obtained through quantitative image analysis of SEM micrographs using ImageJ (Figure S14). Control ZIF‐8 displayed the largest size (1654 ± 384 nm), followed by BSA@ZIF‐8 (947 ± 173 nm) (Figure S14). In contrast, the addition of surfactants during synthesis led to a marked reduction in particle dimensions. Lecithin yielded the smallest particle size (431 ± 72 nm), followed by CTAB (535 ± 98 nm), GMO (770 ± 234 nm), and Triton X‐100 (940 ± 85 nm) (Figures S13 and S14). SEM images showed similar rhombic dodecahedral structures with a rough surface. These results suggest that surfactants, particularly lecithin and GMO, significantly influence nucleation and growth processes, promoting the formation of smaller, more compact MOF structures.

In addition, a 77K N_2_ sorption test was conducted for ZIF‐8 and protein@ZIF‐8 in the presence and absence of surfactants to evaluate the textural properties of composites and the effect of protein encapsulation and surfactants on pore structure (Figure 4f and Figure S15). The data obtained from multipoint Brunauer–Emmett–Teller (BET) and Density Functional Theory (DFT) analyses are summarized in Table S5. Interestingly, the pore width of ZIF‐8 remains constant at approximately 17.2 Å across all samples (Figure S15 and Table S5), which is within the reported literature range and confirms that protein encapsulation does not disrupt the intrinsic pore size or geometry of the framework. The high pore width would potentially reduce the mass transfer resistance of small molecules for biocatalysis applications. The surface area of the pristine ZIF‐8 measured ∼2060 m^2^/g, which confirms the high crystallinity and efficient activation during sample preparation. Upon protein encapsulation, a consistent decrease in surface area is observed across all samples with and without surfactant, indicating successful incorporation of the protein within the porous matrix (Table S5). Interestingly, variations among the surfactant‐assisted composites reveal that different surfactants influence the surface area to varying degrees. For example, BSA‐Lecithin@ZIF‐8 exhibits lower surface areas than BSA@ZIF‐8, which could be due to the stronger dual interactions of lecithin with both protein and the ZIF‐8 interface. This event can potentially enhance protein encapsulation in MOF using lecithin, which is in agreement with the encapsulation efficiency data presented in Figure 4C, and lead to greater pore filling and lower surface areas (Table S5).

Powder x‐ray diffraction (XRD) patterns confirmed the crystalline structure of ZIF‐8 in the BSA@ZIF‐8, both before and after protein encapsulation and in the presence of surfactants (Figure S16a). All BSA@ZIF‐8 samples, with or without surfactants, exhibited diffraction patterns consistent with the characteristic ZIF‐8 framework (Figure S16a). These findings are consistent with SEM, which showed a similar rhombic dodecahedral morphology for both ZIF‐8 and BSA@ZIF‐8 (Figure S13). Thermogravimetric analysis (TGA) curves showed distinct weight loss around 300°C for free BSA and BSA@ZIF‐8, indicating the successful incorporation of BSA into the MOF. The increased char yield from 15% for free BSA to 38% for BSA‐GMO@ZIF‐8 suggests enhanced thermal stability of BSA upon encapsulation (Figure S16b and Table S6). Previous studies have demonstrated that encapsulating biomacromolecules within MOFs can significantly enhance their stability under harsh conditions and extend their storage time [65]. For example, biomimetic mineralization using MOFs has been shown to protect proteins, enzymes, and DNA from denaturation by forming a crystalline exoskeleton under mild, physiological conditions. These protective shells preserve bioactivity even after exposure to extreme thermal and chemical stressors, including boiling in organic solvents such as dimethylformamide and heating up to 80°C, which would normally inactivate free proteins [65]. These findings support the idea that MOF‐based encapsulation can enhance protein stability and function, aligning with our design strategy for surfactant‐mediated protein@MOF assemblies. Attenuated total reflection Fourier transformed infrared spectroscopy (ATR‐FTIR) confirmed the encapsulation of BSA by the appearance of the amide band in 1700−1500 cm^−1^ region (Figure S16c). Additionally, colloidosome‐like assemblies (∼10 µm) were sporadically observed in BSA–GMO@ZIF‐8 and BSA–lecithin@ZIF‐8 samples (Figure S16d). Although these structures were not intentionally targeted and appeared only in rare, isolated instances; their exclusive presence in GMO‐ and lecithin‐containing systems suggests that these surfactants may induce localized interfacial phenomena, potentially consistent with transient Pickering emulsion‐like behavior [63, 68].

**FIGURE 5:**
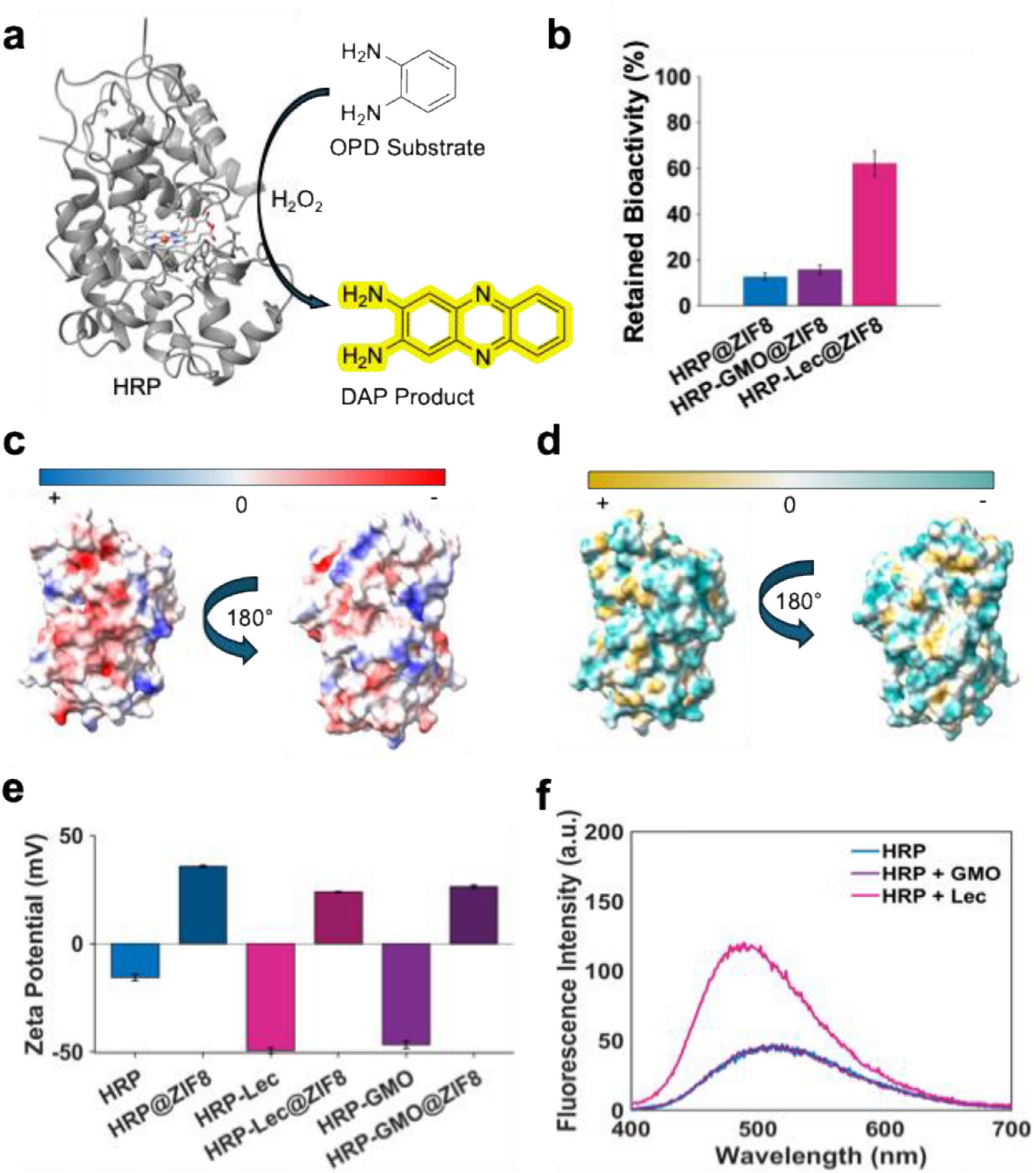
(a) Schematic representation of HRP catalytic oxidation of OPD in the presence of H_2_O_2_, yielding the DAP product. (b) Retained bioactivity (%) of HRP@ZIF‐8, HRP‐GMO@ZIF‐8, and HRP‐Lec@ZIF‐8. (c) Schematic representation of HRP's electrostatic potential, red: negative potential, white: zero, blue: positive potential, PDB ID: 1HCH. (d) Schematic representation of HRP's hydrophobicity distribution, dark cyan for most hydrophilic and dark goldenrod for most hydrophobic, PDB ID: 1HCH. (e) Zeta potential measurements of free HRP, HRP‐surfactants, and the corresponding encapsulated HRP@ZIF‐8 samples. (f) ANS fluorescence emission spectra of free HRP and HRP in the presence of GMO and lecithin.

To assess the transferability of the mechanistic insights derived from the BSA@MOF platform, we applied the surfactant‐driven interfacial design strategy to HRP, one of the most widely used heme‐containing enzymes in biocatalysis applications. BSA and HRP differ in several fundamental physicochemical aspects. BSA is a larger protein (66.5 kDa) in comparison with the HRP (44 kDa). In terms of dimensionality, BSA with unit cell size of 21.7 nm × 4.4 nm × 14.3 nm (extracted from the x‐ray crystal structure, PDB: 4F5S) adopts an elongated, ellipsoidal conformation composed of three domains [69, 70, 71], whereas HRP with unit cell size of 4 nm × 6.7 nm × 11.7 nm (extracted from the x‐ray crystal structure, PDB: 1HCH) is more compact with a globular structure centered around a heme cofactor (Figure 5a) [72]. These differences indicate that the two proteins occupy distinct size and shape regimes. BSA is known to be relatively flexible and conformationally adaptable, consistent with its role as a transport protein with multiple ligand‐binding sites [69, 73]. In contrast, HRP is a more rigid enzyme with a well‐defined tertiary structure required to maintain catalytic activity [74]. With respect to hydrophobicity, BSA contains large hydrophobic binding pockets and exhibits pronounced amphiphilic character, enabling strong interactions with hydrophobic ligands (Figure 1c,d) [69]. HRP, while also possessing localized hydrophobic regions particularly near the active site, presents a comparatively more hydrophilic external surface overall (Figure 5d) [75, 76]. Thus, the two proteins differ in both the extent and distribution of hydrophobic domains. Despite these differences, the proposed nucleation mechanism is governed primarily by surface charge distribution and the presence of the surface residues capable of coordination (Figures 1c and 5c), rather than protein identity. Importantly, both BSA and HRP exhibit similar surface charge characteristics under the synthesis conditions (Figures 1c and 5c). BSA has an isoelectric point (pI) of ∼4.7–4.9, while HRP (isoform VI from *Armoracia rusticana*) has a calculated pI of ∼5.67 (ExPASy), meaning that both proteins are net negatively charged at pHs above their pI, which was the case in our synthetic environment. This similarity in the electrostatic environment supports comparable interactions with metal precursors. In addition, both proteins expose amino acid residues such as histidine, aspartate, and glutamate, which can coordinate with Zn, providing nucleation sites for MOF growth [77, 78]. Furthermore, both proteins exhibit heterogeneous surface charge distributions, enabling localized interactions with precursors and supporting the general ability of this platform to encapsulate proteins (Figures 1c,d and 5c,d).

We demonstrated that the addition of lecithin and GMO increased the surface negative charge of the HRP, resulting in the encapsulation of HRP in MOF (Figure 5e). The positive charge of the HRP‐surfactant@ZIF8 composites reflects the successful encapsulation of HRP in the MOF. HRP‐Triton and HRP‐CTAB with zeta potentials of −4.0 ± 1.5 and +19 ± 7, respectively, didn't form any HRP@MOF composites upon the addition of MOF precursors, potentially due to the lack of sufficient negative surface charge.

We evaluated the retained enzymatic activity of HRP encapsulated in ZIF‐8 under different surfactant conditions (Figure 5a,b). In the bioactivity assay, hydrogen peroxide was provided as the oxidant, and o‐phenylenediamine (OPD) was used as the substrate (Figure 5a). HRP catalyzes the oxidation of OPD by hydrogen peroxide, a prototypical peroxidase reaction that involves O─O bond activation followed by oxidation of aromatic C─H bonds on OPD, producing 2,3‐diaminophenazine (DAP), a yellow oxidized product with strong absorbance at 418 nm. Fresh free HRP was used as a baseline with 100% activity. Direct encapsulation into ZIF‐8 without surfactants retained only ∼13% of native activity, consistent with prior reports due to potential restricted substrate accessibility. In contrast, surfactant‐assisted encapsulation markedly improved activity retention. HRP–lecithin@ZIF‐8 preserved ∼62% of native activity, approximately six‐fold improvement compared to HRP@ZIF‐8 without surfactant (Figure 5b).

Moreover, initial rates of activity were assessed to determine the Michaelis–Menten parameters for free HRP and HRP@ZIF‐8 and HRP‐Lec@ZIF8 composites. As shown in Figure S17, free HRP exhibits the highest catalytic rate with a V_max_ of 71.5 µM s^−^^1^. In comparison, HRP@ZIF‐8 shows a substantially reduced V_max_ (0.2 µM s^−^^1^). Remarkably, lecithin modulation enhances the V_max_ of HRP‐Lec@ZIF‐8 to 15.50 µM s^−^^1^, corresponding to a ∼15‐fold improvement relative to HRP@ZIF‐8. This trend is further reflected in the turnover number (*k*_cat_) (Table S7). While free HRP represents the intrinsic upper benchmark of enzymatic activity (795 s^−1^), HRP‐Lec@ZIF‐8 exhibits a *k*_cat_ of 172 s^−1^, representing nearly a 60‐fold increase compared to HRP@ZIF‐8 (3 s^−1^) (Table S7). These results indicate that lecithin plays a critical role in preserving enzymatic function during MOF encapsulation (Figure S17 and Table S7). This enhancement can be attributed to two complementary effects. First, ANS fluorescence measurements suggest that lecithin promotes the hydrophobicity on the protein surface (Figure 5f), improving compatibility between HRP and the hydrophobic microenvironment within ZIF‐8 pores and thereby helping preserve the native‐like tertiary structure [79]. Second, lecithin influences MOF formation itself, leading to smaller final particle sizes (∼400 nm compared to ∼900 nm for HRP@ZIF‐8) (Figure S13). This reduction in crystal size is expected to improve substrate accessibility and product diffusion due to shorter path lengths. Moreover, decreasing particle size increases the specific surface area, thereby improving the accessibility of active sites and facilitating more efficient interaction between the enzyme and substrate, further contributing to the observed increase in catalytic efficiency. These results highlight the beneficial role of surfactants in preserving enzyme functionality and support the promise of this strategy for application in biocatalysis.

For HRP in the presence of GMO, the absence of a significant change in ANS fluorescence intensity indicates that the enzyme's native surface hydrophobicity remains largely unaltered upon GMO exposure (Figure 5f). This behavior suggests that HRP preserves its native structural microenvironment in the presence of GMO. In contrast to BSA‐lecithin system, the HRP‐lecithin system shows a dramatic increase in ANS fluorescence intensity accompanied by a blue shift (Figure 5f), which is indicative of ANS being transferred into a more hydrophobic and less polar environment. The observed features of ANS, a blue shift of fluorescence emission maxima and the increase of fluorescence intensity, are generally attributed to the hydrophobicity of a binding site and the restricted mobility of ANS [56]. Unlike BSA, HRP is a smaller, more rigid, heme‐containing protein with limited internal hydrophobic cavities available. Lecithin in this case is more likely to act as a membrane‐mimetic scaffold, assembling on or near the protein surface rather than occupying deep internal binding pockets. This can create localized hydrophobic domains (lecithin‐rich clusters) that promote ANS partitioning at the protein–lecithin interface. The blue shift suggests that ANS experiences a more apolar microenvironment than in native HRP, consistent with insertion into lecithin‐associated hydrophobic regions or interfacial binding sites rather than aqueous exposure. Therefore, the contradictory behavior can be summarized as a protein‐dependent balance between competitive binding inside pre‐existing hydrophobic cavities (dominant in BSA) and formation of new lipid‐induced hydrophobic microdomains at protein surfaces (dominant in HRP). Therefore, lecithin effectively reduces ANS‐accessible binding sites in BSA, whereas in HRP it reorganizes the local environment to create favorable ANS‐binding hydrophobic interfaces; hence, HRP‐lecithin is more compatible with the intrinsic hydrophobic character of ZIF‐8, resulting in enhanced enzyme activity compared with the HRP@ZIF‐8 (Figure 5b and Figure S17).

We also tested the stability of HRP‐lecithin@ZIF‐8 and HRP‐GMO@ZIF‐8 in comparison to free HRP in the presence of a protease, trypsin, as a harsh condition. We treated the HRP@MOF with trypsin for 2 h and measured the initial rate of the enzyme as well as the product formation capability of the enzyme in 10 min period as an indication of bioactivity in comparison with free HRP (Figure S18). After treating the HRP@MOF with protease, we digested the MOF structure with EDTA to release the HRP for performing the bioactivity tests. Following trypsin treatment, enzymes extracted from HRP@ZIF‐8 and HRP–GMO@ZIF‐8 exhibited higher initial substrate‐to‐product conversion rates compared to free HRP, with rates of ∼0.16 µM s^−^^1^ vs. 0.12 µM s^−^^1^, respectively, corresponding to ∼30% greater retention of catalytic activity (Figure S18b). These findings indicate that the MOF provides a protective effect, enhancing the activity retention of the encapsulated enzyme. Furthermore, in a 10‐min bioactivity assay, HRP extracted from HRP‐GMO@ZIF8 exhibited higher overall bioactivity (more final product formation), showing roughly 30% more activity compared to free HRP (Figure S18c). In contrast, HRP from HRP@ZIF8 demonstrated nearly the same bioactivity as the free HRP.

We envision that protein‐GMO@MOF and protein‐lecithin@MOF composites could potentially be used to stabilize the membrane proteins by imitating their dynamic interactions with lipids [41]. Thus, they show strong potential as versatile alternatives to lipid‐based nanodiscs for stabilizing membrane proteins. This is especially important given the ongoing challenge of preserving membrane protein integrity in cell‐free environments, where native lipid bilayers are absent and denaturation is common [80, 81]. Unlike traditional nanodiscs, which often exhibit limited long‐term stability and incompatibility with solid‐state or heterogeneous environments [82, 83, 84, 85], protein@MOF composites with integration of lipid‐based nonionic surfactants may offer a more robust and adaptable platform. This has significant implications for pharmaceutical research on sensitive proteins, where approximately 60% of drug targets are membrane‐associated proteins [42, 43, 86, 87, 88]. Developing stable, bioinspired matrices that preserve protein conformation and improve catalytic efficiency could open new pathways for biocatalysis and biosensing.

## 3 Conclusions

In summary, this study establishes a mechanistic framework for understanding how surfactant‐mediated interfacial design governs the encapsulation of proteins within metal–organic frameworks. By employing a combination of biochemical assays, spectroscopy, microscopy, and MD simulations, we reveal how surfactants modulate protein–MOF interactions through alterations in electrostatic potential, hydrophobicity, and protein surface presentation. Our findings show that lipid‐based nonionic surfactants, particularly GMO and lecithin, promote favorable interfacial environments that preserve protein secondary structure, enhance encapsulation efficiency, and catalytic efficiency. Importantly, domain‐specific binding patterns observed in simulations support a model where localized, stabilizing interactions between surfactant molecules and protein residues guide MOF nucleation and particle organization. These insights contribute to a clearer understanding of how proteins behave at the interface and underscore the role of molecular design in optimizing protein@MOF formation and function. Beyond advancing the fundamental mechanistic understanding of the assembly of biomolecular inorganic materials, this work offers a versatile and practical strategy for engineering such materials with improved retention of structure and function compared to traditional techniques based on nanodiscs. Thus, it opens new doors to future applications in biocatalysis, biosensing, membrane protein stabilization, and drug delivery.

## Author Contributions

**Ruibin Liang**: investigation, funding acquisition, methodology, validation, visualization, writing – review and editing, software, formal analysis, data curation, resources, writing – original draft. **Carlie Coerver**: formal analysis, writing – review and editing. **Raheleh Ravanfar**: investigation, conceptualization, methodology, software, data curation, supervision, resources, project administration, formal analysis, validation, visualization, writing – review and editing, writing – original draft, funding acquisition. **Mohammad Khavani**: data curation, formal analysis, investigation, methodology, visualization, writing – review and editing. **Ehsan Rashidniyaghi**: data curation, formal analysis, investigation, methodology, visualization, writing – review and editing.

## Conflicts of Interest

The authors declare no conflicts of interest.

## Supporting information




**Supporting File**: advs76011‐sup‐0001‐SuppMat.docx

## Data Availability

The supplementary information, raw data and custom analysis scripts are available upon request from the corresponding author.
