# Supplementary material for: Mechanistic Understanding of Protein–MOF Integration Through Surfactant‐Driven Interfacial Design
Source: Adv Sci (Weinh). 2026 Jun 9;13(52):e76011. doi: 10.1002/advs.76011 (PMC13336444; doi:10.1002/advs.76011)
Supplement: Supplementary file 1 [file ADVS-13-e76011-s001.docx]

Supporting Information

Mechanistic Understanding of Protein–MOF Integration through Surfactant-Driven Interfacial Design

Ehsan Rashidniyaghi, Mohammad Khavani, Carlie Coerver, Ruibin Liang*, Raheleh Ravanfar*

**MATERIALS AND METHODS**

Materials

Bovine Serum Albumin (BSA) was purchased from Fisher Bioreagent, NJ, USA. Zinc nitrate 6-Hydrate was purchased from Ward’s Science, ON, Canada. Hexadecyltrimethylammonium bromide (CTAB) and 2-methylimidazole were obtained from Sigma-Aldrich, MO, USA. Glycerol monooleate (GMO) was purchased from Spectrum Chemical MFG Corp, Gardena, CA, USA. Soy lecithin was obtained from Velona Incorporate, IL, USA. Triton X-100 was obtained from ThermoFischer Scientific, MA, USA. Bradford 1X dye reagent was purchased from Bio-Rad, California, USA. All the chemicals were used as received without further purification.

Experimental Methods

Synthesis of protein@MOF and MOF

Protein@MOFs and MOFs were synthesized using previously reported coprecipitation methods,^[1]^ at room-temperature in aqueous-phase with a Zn^2+^: 2-methylimidazole (HmIM) molar ratio of 1:60. In a typical procedure, aqueous solutions of zinc nitrate hexahydrate (Zn(NO_3_)_2_·6H_2_O) and HmIM were mixed to initiate crystallization of MOF. For protein@MOFs, 10 µM Bovine Serum Albumin (BSA) as a model protein was added to HmIM, followed by the addition of zinc nitrate to initiate encapsulation. The final mixture maintained a Zn^2+^:HmIM ratio of 1:60. Surfactants, including GMO, CTAB, lecithin, and Triton X-100, were introduced prior to MOF mineralization to modulate protein interfacial properties to an optimized final surfactant concentration of 70 µM based on encapsulation efficiency measurements. Briefly, the encapsulation efficiency of BSA@ZIF-8 was evaluated at three surfactant concentrations (70, 150, and 300 µM) across different surfactant systems (Triton X-100, GMO, lecithin, and CTAB). In most cases, 70 µM resulted in the highest or comparable EE% relative to higher concentrations. Notably, increasing surfactant concentration to 150 and 300 µM led to either a plateau or a decrease in EE%, particularly for Triton X-100 and lecithin systems (Figure S19).

To confirm the transferability of the optimized conditions from BSA to HRP, the concentration screening for lecithin was done in the synthesis process across various concentrations (10, 70, 500, and 5000 µM), showing that the highest EE% at 70 µM was applicable to HRP system as well (Figure S20).

We also optimized the effect of MOF precursor concentration on encapsulation efficiency (Figure S21). Briefly, we screened two different concentrations of MOF precursors, including Zn and HmIM, (Zn: 2 mM, 2MIM: 120 mM) and (Zn: 20 mM, 2MIM: 1200 mM). These results show that increasing precursor concentration by 10 folds leads to 15% higher encapsulation efficiency (Figure S21).

Encapsulation Efficiency using Bradford Assay

Dried sample (1 mg) was dispersed in 5 µL water, following by the addition of 250 µL Bradford reagent was added according to the Bio-Rad protocol. The absorbance at 595 nm was used to calculate the encapsulation efficiency. The standard curve was created according to microplate standard assay from the Bio-Rad for Bradford assay for the range of 125-2000 µg/ml.

Encapsulation efficiency (EE%) obtained from the Bradford assay reflects the fraction of protein captured from the supernatant during the formation of protein@MOF, calculated as following:

$$Encapsulation Efficiency (\%)=(\frac{Protein input-Protein in supernatant}{Protein input})\times100$$

Protein Loading according to the Elemental Analysis

Following synthesis, samples (BSA@ZIF-8, BSA–surfactant@ZIF-8, and surfactant@ZIF-8 controls) were collected by centrifugation at 27,000 ×g for 10 min and washed three times with Milli-Q water to remove unreacted species. For ZIF-8-only samples, an additional centrifugation step of 20 min was performed to ensure complete pelleting. Finally, the washed samples were dried at room temperature for 48 h prior to collection and subsequent elemental analysis.

To obtain quantitative results, we employed CHNS elemental analysis, which provides accurate protein loading within MOFs and directly measures the protein content retained in the final dried MOF composite. Protein loading is therefore sensitive to both protein incorporation and the amount of MOF formed. In particular, sulfur was used as a selective elemental marker, as it is present in the protein (from cysteine and methionine residues) but absent in the ZIF-8 framework.

Based on amino acid composition analysis of BSA extracted from ExPASy ProtParam (PDB: 4F5S; UniProtKB: P02769), the sulfur content of BSA is approximately 2 wt%. The BSA loading in BSA@MOF (wt%) was determined using the following formula:

$$wt\% BSA in BSA@MOF=(\frac{Measured S (wt\%) in BSA@MOF}{S Fraction in BSA (wt\%)})\times100$$

Protein Surface Hydrophobicity Assessment

The exposed hydrophobicity of native and surfactant-modulated proteins was evaluated using an ANS fluorescence assay. Stock solutions of BSA and HRP were prepared in aqueous solution and used in the samples with final concentrations of 10 μM. Lecithin and GMO were used with a final concentration of 700 μM. ANS was prepared separately in the same condition and used with a final concentration of 250 µM. For each measurement, protein samples (BSA, BSA-GMO, BSA-Lec, HRP, HRP-GMO, and HRP-Lec) were incubated with ANS under identical conditions for 30 minutes to allow probe binding to hydrophobic regions exposed on the protein surface. Then, the fluorescent measurement was conducted. The excitation wavelength was set at 360 nm, and emission spectra was recorded from 400 to 700 nm. Instrument parameters were optimized separately for each protein system: BSA samples were recorded at a photomultiplier tube (PMT) voltage of 680 V with a slit width of 2.5 nm, while HRP samples were recorded at 840 V with a slit width of 5 nm to ensure adequate signal intensity. Fluorescence spectra were recorded at room temperature using standard quartz cuvettes with a 1 cm path length. Appropriate baseline corrections were applied by subtracting buffer blanks measured under identical conditions.

Scanning Electron Microscopy and Elemental Analysis

Surface morphology was investigated by scanning Electron Microscopy (SEM) using a Zeiss crossbeam 540 FIB-SEM equipped with Oxford energy-dispersive X-ray spectroscopy (EDS) system from ZEISS, 07745 Jena, Germany for elemental analysis. The SEM images were collected at 5 kV accelerating voltage and 5.9 mm working distance with side mount secondary electron detector. Samples were resuspended in water and dropped onto aluminum sample stubs and were observed after drying.

Powder X-ray diffraction (PXRD)

Crystalline structure of the protein@MOF was investigated using PXRD. Samples were dried using a Savant Speed Vac Plus SC110A from Savant Instruments Inc. Farmingdale, New York, USA. The samples were then placed on a sample disk and PXRD patterns were recorded at a 5°/min scanning speed and 5−60° diffraction angle by a Rigaku MiniFlex II Powder X-Ray Diffractometer with Cu Ka radiation set at 30 kV and 15 mA from Rigaku Americas Corporation, 9009 New Trails Drive, The Woodlands, TX, USA.

Thermal Gravimetric Analysis (TGA)

Thermal stability of the samples was investigated using TGA. The samples (10 mg) were placed on a sample pan and heated in a Nitrogen atmosphere from 25 °C to 800 °C at a rate of 10 °C/min using a Shimadzu DTG-60H simultaneous DTA-TG apparatus from Shimadzu Corporation, Kyoto, Japan. The char yield was calculated according to the weight loss (%).

Growth Kinetics Measurements

Time-resolved size growth of the protein@MOF composites was investigated by dynamic light scattering (DLS) mode of Malvern Zetasizer pro from Malvern Panalytical Ltd., Grovewood Road, Malvern, Worcestershire, WR14 1XZ, United Kingdom. This study was performed using disposable plastic cuvettes provided by Malvern. The study was carried out through synthesis of protein@MOF in the presence and absence of surfactants with a final protein concentration of 1 µM. Data collection initiated immediately after mixing all components. Measurements were conducted for 10 cycles with optimized instrument settings, positioning at the center, attenuation of 5, 15 runs, and run duration of 0.86 seconds. All surfactant solutions were prepared in 50% ethanol. Experiments were done in triplicates. The size growth was recorded for 300 seconds, and the growth was fitted to the exponential equation using MATLAB to calculate the initial rates for the protein@MOF in the presence and absence of different surfactants.

Zeta (ζ) Potential Analysis

Zeta potential analysis was conducted using the static light scattering (SLS) mode of Malvern Zetasizer Pro using folded capillary zeta cell (DTS1070) cuvettes supplied by Malvern. The final concentration of protein in this study was 10 µM.

Circular Dichroism Spectroscopy (CD)

Secondary structure of 1 µM BSA in the presence of surfactant solutions was investigated using a J-815 CD, Tokyo, Japan. Secondary structure calculations were carried out with BeStSel.^[2]^ The raw ellipticity data was in millidegrees with the path length of 0.1 cm. Single spectrum calculation was conducted on the data on 195-250 nm wavelength range with a scale factor of 2.

Attenuated total reflection Fourier transformed infrared spectroscopy (ATR-FTIR)

ATR-FTIR was performed on a Nicolet iS10 FT-IR with a Smart iTX ATR sampling accessory from ThermoFisher Scientific, Thermo Electron Scientific Instruments LLC, Madison, WI, USA.

Ultraviolet–visible (UV-vis) Spectrophotometry

UV-vis spectrophotometry was conducted in the range of 190-900 nm, with 1.0 nm data interval, and 0.004 s averaging time on an Agilent Cary UV-Vis Compact Peltier from Agilent Technologies, Mulgrave, Australia.

Brunauer–Emmett–Teller (BET) Analysis

Surface area analysis was performed using N_2_ adsorption-desorption isotherms at 77K with a Quantachrome® ASiQwin™ analyzer. Prior to measurements, samples were degassed under vacuum at 100°C for 12h to eliminate adsorbed gases and moisture. The specific surface area of the samples was determined using the Brunauer–Emmett–Teller (BET) method. The BET equation was applied in the relative pressure range (P/P₀) of 0.05–0.50, where the isotherms showed linearity. The surface area was calculated based on the measured adsorption data. Pore size distribution and pore volume were further analyzed using Density Functional Theory (DFT) applied to the N_2_ adsorption-desorption isotherms. The analysis was carried out using N_2_ at 77 K on carbon (cylindrical pores, QSDFT adsorption branch). Model selection was based on the expected pore shape determined from SEM.

Enzyme activity Assay

HRP@MOF was resuspended using brief sonication followed by vortexing to ensure uniform dispersion. To initiate the enzymatic reaction, hydrogen peroxide (H_2_O_2_) and o-phenylenediamine (OPD) as substrate were added. After incubation at room temperature for 15 minutes, the absorbance of the oxidized product was measured at 418 nm to assess the retained peroxidase activity of the encapsulated enzyme. **Michaelis–Menten kinetic parameters were determined by measuring HRP-catalyzed product formation in an aqueous buffer at room temperature.** The concentration of HRP was adjusted to 450 nM in both the free-enzyme and protein@MOF composites, and H_2_O_2_ was varied from 0.1 to 20 mM in the presence of 1 mM OPD. Initial reaction rates were calculated from the first 30 s of product formation and fitted to the Michaelis–Menten equation.

Protein@MOF Stability Assessment in the presence of Protease

HRP@ZIF-8, HRP-GMO@ZIF-8, and HRP-Lecithin@ZIF-8 were synthesized by mixing appropriate amounts of reagents to obtain final concentrations of 1 µM HRP, 700 µM surfactant (where applicable), 249 mM HmIm, and 4.15 mM Zn²⁺ (HmIm/Zn = 60). The reaction mixtures were allowed to proceed for 12 h at room temperature. The resulting particles were collected by centrifugation at 14,000 rpm for 15 min and washed three times with DI water to remove unreacted species. The washed pellets were resuspended in 1 mL of Tris–HCl buffer (0.1 M, pH 8.0) containing 6 mg/mL trypsin and incubated for 2 h at 37°C. Following incubation, samples were washed thoroughly with DI water to remove residual trypsin. To extract encapsulated HRP, the samples were treated with 0.03 M EDTA and incubated at room temperature for 30 min. Rapid clarification of the solution within the first few minutes indicated effective digestion of the ZIF-8. After digestion, 4 mL of DI water was added to each sample. The solutions were then subjected to centrifugal filtration using 30 kDa molecular weight cutoff filters at 4500 rpm for 10 min. Final filtrate volumes were reduced to approximately 200 µL. For HRP-Lecithin@ZIF-8 samples, incomplete filtration was observed due to filter clogging, likely caused by residual particulates. To address this, samples were pre-filtered using 0.22 µm PVDF filters prior to downstream analysis. The concentration of extracted HRP was determined using UV–Vis spectroscopy by measuring absorbance at 403 nm. The corresponding molar concentration of HRP was calculated to quantify enzyme recovery after filtration. To evaluate enzymatic activity, all samples were normalized to a final HRP concentration of 20 nM. The reaction was initiated by adding 1 mM OPD and 100 µM H₂O₂. The formation of the oxidized product was monitored spectrophotometrically at 5 s intervals over a period of 10 min at wavelength range of 200-800 nm.

Computational Methods

Molecular dynamics (MD) simulations were carried out to investigate the interactions between BSA and GMO. These simulations helped identify the protein residues that interact most strongly with the surfactant molecules. The structure of BSA was obtained from the Protein Data Bank (PDB ID: 4F5S). BSA was placed at the center of a cubic simulation box (117 × 117 × 117 Å^3^), and seven GMO molecules were randomly placed around the protein using PACKMOL software,^[3]^ ensuring that all atoms of each GMO maintained a minimum distance of 5 Å from every protein atom. Periodic boundary conditions were applied in all directions throughout the simulations. To mitigate the sensitivity of the simulation results on the initial positions of the surfactant, six different simulation systems were prepared, each with a distinct arrangement of GMO molecules around the BSA. Additionally, a separate simulation without any GMO molecules was performed to examine the dynamical behavior of BSA in the absence of surfactants. Na⁺ ions were added to neutralize the net charge of the systems. In a separate series of simulations (six in total), a 0.15 M concentration of NaCl was added to test the effects of ion concentration on protein–surfactant interactions. Additionally, to investigate the effect of GMO concentration on its interactions with BSA, an additional simulation setup was constructed containing 100 GMO molecules in the presence of 0.15 M NaCl. The CHARMM-GUI website interface^[4]^ was utilized to solvate the BSA-GMO complex with water molecules and ions and generate the input files for the simulations.

First, all systems were relaxed through geometry optimization. Then, a 250 ps simulation was performed in the constant NVT ensemble at 300 K temperature. The temperature of the simulation setups was controlled using the Langevin thermostat with a friction coefficient of 1 ps⁻¹. In the next step, for each system, a 10 ns MD simulation was conducted in the constant NPT ensemble at 300 K temperature and 1 atm pressure, which was treated as the equilibration simulation. Then, each system was subject to a 300 ns equilibration in the same constant NPT ensemble, which was treated as the production simulation. A 2 fs timestep was employed during the NPT simulations, with constraints applied to the lengths of all covalent bonds involving hydrogen atoms. The snapshots were saved in 0.1 ns time intervals. The system pressure was controlled by the Langevin piston Nose–Hoover method.^[5]^ MD simulations were conducted using the NAMD software package.^[6]^ The protein and surfactant molecules were treated with the CHARMM36m force field.^[7]^ Water molecules were described using the TIP3P model.^[8]^ Electrostatic interactions were treated with the particle mesh Ewald method,^[9]^ and a 12 Å cutoff was applied for van der Waals interactions. The solvent accessible surface area (SASA) values of the protein in the presence and absence of the surfactant molecules were calculated using VMD 1.9.3.^[10]^ Furthermore, to determine the spatial distribution of the GMOs’ head groups around the protein, we constructed occupancy maps averaged over all six independent MD simulations without NaCl. These maps were generated based on the center of mass (COM) positions of all surfactant head groups throughout all MD trajectories, after aligning the protein backbone in each frame to the initial one as the reference structure. The analysis was carried out using VMD,^[10]^ and the data were rendered as a three-dimensional isosurface to visualize the spatial distribution of the surfactant head groups with minimum occupancy 0.005.

To identify the protein residues with strong binding interactions with GMOs, we calculated the conformational factor for each residue ($\boldsymbol{P}_{\boldsymbol{i}}$, with $\boldsymbol{i}$ being the residue number) that reflects how often each residue comes into contact with the surfactant’s head group, since these interactions are more likely to occur at binding sites. The following method was employed to calculate the $\boldsymbol{P}_{\boldsymbol{i}}$’s: whenever any heavy atoms of the head group of a GMO molecule came within 5 Å of any heavy atom of residue *i*, it was counted as a contact.^[11]^ For each residue, the total number of these contacts was recorded throughout all saved frames in all trajectories. Then, we calculated the average number of contacts per residue using the formula $\boldsymbol{\langle n\rangle= (\sum}\boldsymbol{n}_{\boldsymbol{i}}\boldsymbol{)/N}$, where $\boldsymbol{n}_{\boldsymbol{i}}$ is the number of contacts with the $\boldsymbol{i}$th residue and $\boldsymbol{N}$ is the total number of residues in BSA. The conformational factor $\boldsymbol{P}_{\boldsymbol{i}}$ for each residue was defined as $\boldsymbol{P}_{\boldsymbol{i}}\boldsymbol{=}\boldsymbol{n}_{\boldsymbol{i}}\boldsymbol{/\langle n\rangle,}$ , which categorizes the strength of residue-GMO interactions: residues with $\boldsymbol{P}_{\boldsymbol{i}}$ $\boldsymbol{\approx}$ 1 interact with the GMO at an average level; residues with $\boldsymbol{P}_{\boldsymbol{i}}\boldsymbol{\gg1}$ and $\boldsymbol{P}_{\boldsymbol{i}}\boldsymbol{\ll1}$ indicate higher or lower affinity with GMO than the average over all residues in the protein.

Figure S1. Schematic diagram illustrating specific binding interactions of surfactants at the protein–MOF interface, including potential hydrogen bonds (black dots) and hydrophobic interactions (red dots). The protein surface is colored by hydrophobicity pattern (dark cyan = most hydrophilic; dark goldenrod = most hydrophobic), using UCSF ChimeraX software, PDB ID: 4F5S. Hydrophobic interactions occur between the non-polar side chains of surface-exposed amino acids and the hydrophobic tails of surfactants. These tails may also interact with the hydrophobic interior surfaces of the ZIF-8 MOF. In parallel, the hydrophilic headgroups of surfactants can form hydrogen bonds with polar or charged amino acid residues on the protein surface, contributing to interfacial stabilization.

Figure S2. Secondary structure determination of BSA control and BSA in the presence of various surfactants.

Figure S3. The calculated $\boldsymbol{P}_{\boldsymbol{i}}$ values for each residue of BSA across six independent MD simulations without NaCl concentration. The residues with $\boldsymbol{P}_{\boldsymbol{i}}\boldsymbol{\gg1}$ interact with the GMO molecules with above-average frequencies.

Figure S4. The calculated $\boldsymbol{P}_{\boldsymbol{i}}$ values for each residue of BSA across six independent MD simulations in the presence of 0.15 M NaCl. The residues with $\boldsymbol{P}_{\boldsymbol{i}}\boldsymbol{\gg1}$ interact with the GMO molecules with above-average frequencies.

**Figure S5.** Stability and flexibility analysis of BSA in the presence and absence of GMO. (a) Backbone RMSD of BSA over 300 ns in the presence of GMO and 0.15 M NaCl, shown for six independent simulations along with the control system without GMO. (b) Backbone RMSD under the same conditions in the absence of NaCl. In both cases, the RMSD increases during the initial equilibration period and then stabilizes, indicating that the systems reach equilibrium without significant structural drift. (c) The calculated RMSF of BSA in the presence of GMO and NaCl. (d) The calculated RMSF in the absence of NaCl. The RMSF profiles are consistent across independent simulations, with higher flexibility observed mainly in terminal regions, as expected for BSA.

Figure S6. Size growth analysis using dynamic light scattering (DLS) for the formation of BSA@MOF in the presence of various surfactants in 250 seconds.

Figure S7. Growth kinetics with exponential fits for the formation of BSA@MOF in the presence of various surfactants to calculate initial rates.

Figure S8. Effect of surfactants on ZIF-8 crystallization in the absence of proteins

Figure S9. The schematic of Bradford assay, showing the interaction of Coomassie Brilliant Blue (CBB) with the protein’s positive residues and formation of the colored complex with maximum absorbance at 595 nm.

Figure S10. CD spectra of BSA in the presence of MOF precursors, zinc and HmIM, prior to MOF formation.

Figure S11. Elemental analysis of a) ZIF-8 in water, b) BSA@ZIF-8, c) BSA-GMO@ZIF-8, d) BSA-lecithin@ZIF-8, e) BSA-TritonX100@ZIF-8, and f) BSA-CTAB@ZIF-8 composites.

Figure S12. Scanning electron microscopy (SEM) image of rhombic ZIF-8 formed in water, scale bar: 10 µm.

Figure S13. SEM images of rhombic BSA@MOF crystals formed in the presence of different surfactants. Scale bars: 2 µm.

Figure S14. Quantitative image analysis of SEM micrographs using ImgageJ. Scale bars: 2 µm.

Figure S15. Density functional theory (DFT) pore size distribution detected with N_2_ adsorption and desorption at 77 K for ZIF-8 in comparison with the BSA@ZIF-8 in the presence and absence of various surfactants.

**Figure S16.** a) PXRD patterns of BSA@MOF composites showing consistent crystallinity in the presence of various surfactants. b) TGA curves of free BSA and BSA@MOF composites synthesized with different surfactants. c) ATR-FTIR spectra of protein@MOF composites formed in the presence of various surfactants. d) Formation of hollow colloidosomes assembled from individual rhombic BSA@MOF particles in the presence of GMO and lecithin, scale bar: 2 µm.

Figure S17. Initial rates of ADP formation versus H_2_O_2_ concentration to calculate Michaelis–Menten kinetic parameters.

Figure S18. Activity assay and bioactivity analysis of free and encapsulated HRP after treatment with protease. (a) UV–vis spectra recorded over 10 min for the HRP catalyzed oxidation of OPD using 1 mM OPD, 100 μM H2O2, and 10 nM HRP. Spectra are shown for free HRP, HRP@ZIF-8, and HRP-GMO@ZIF-8. (b) Initial reaction rates derived from the activity assay. (c) Relative bioactivity expressed as the final product yield normalized to free HRP.

Figure S19. Optimization of surfactant concentrations based on encapsulation efficiency.

Figure S20. Optimization of HRP encapsulation efficiency in ZIF-8 in the presence of lecithin.

Figure S21. Optimization of HRP encapsulation efficiency in ZIF-8 using different concentrations of MOF precursors.

Table S1. Measured pH values of precursor solutions before and after addition of HmIM. The addition of HmIM results in a consistent alkaline environment (pH ≈ 11) across all systems. Values are reported as mean ± 0.03 (n = 3)

| Sample | pH (before HmIm) | pH (after HmIm) |
| --- | --- | --- |
| BSA | 7.56 | 10.96 |
| BSA-Triton | 7.51 | 10.94 |
| BSA-CTAB | 6.44 | 10.92 |
| BSA-GMO | 7.48 | 10.96 |
| BSA-Lecithin | 6.98 | 10.95 |

Table S2. Residues with top 20 highest $\boldsymbol{P}_{\boldsymbol{i}}$ values averaged over six independent simulations with and without NaCl concentration. The residues with high $\boldsymbol{P}_{\boldsymbol{i}}$ values in both setups are highlighted in bold text.

| $\boldsymbol{P}_{\boldsymbol{i}}$ rank number | 0.15 M NaCl | | 0.00 M NaCl | |
| --- | --- | --- | --- | --- |
|  | Residue | $\boldsymbol{P}_{\boldsymbol{i}}$ | Residue | $\boldsymbol{P}_{\boldsymbol{i}}$ |
| 1 | TYR451 | 9.396 | ARG435 | 16.727 |
| 2 | ARG435 | 9.16 | TYR451 | 15.885 |
| 3 | LEU115 | 7.673 | LYS439 | 15.096 |
| 4 | ALA324 | 7.581 | THR421 | 14.783 |
| 5 | THR231 | 7.478 | PHE205 | 14.708 |
| 6 | LYS439 | 7.441 | ALA324 | 13.541 |
| 7 | GLU186 | 7.285 | GLU186 | 13.169 |
| 8 | ASP323 | 7.223 | LEU462 | 13.131 |
| 9 | LYS211 | 7.197 | ARG208 | 12.904 |
| 10 | LYS116 | 6.981 | ASP323 | 12.899 |
| 11 | PRO113 | 6.661 | THR231 | 12.839 |
| 12 | LYS187 | 6.576 | PHE227 | 12.353 |
| 13 | PRO117 | 6.486 | VAL432 | 11.392 |
| 14 | LEU112 | 6.475 | LYS187 | 11.29 |
| 15 | PHE227 | 6.078 | THR466 | 11.122 |
| 16 | ALA489 | 5.982 | THR190 | 11.101 |
| 17 | GLU478 | 5.912 | LYS350 | 10.637 |
| 18 | LYS350 | 5.638 | LYS204 | 10.545 |
| 19 | PHE205 | 5.469 | VAL468 | 10.415 |
| 20 | SER479 | 5.465 | CYS447 | 10.226 |

Table S3. Kinetics results of protein@MOF growth in the presence of various surfactants.

| Parameters | | No Surfactant | GMO | Lecithin | Triton X-100 | CTAB |
| --- | --- | --- | --- | --- | --- | --- |
| A (nm) | Repeat 1  Repeat 2  Repeat 3 | 1000.00  760.89  600.00 | 1505.16  1562.00  1371.27 | 2154.41  2005.88  2005.28 | 994.19  1098.24  860.77 | 342.00  250.00  315.02 |
| K (1/s) | Repeat 1  Repeat 2  Repeat 3 | 0.03  0.03  0.04 | 0.02  0.02  0.02 | 0.01  0.02  0.02 | 0.02  0.03  0.02 | 0.04  0.03  0.03 |
| AK (nm/s) | Repeat 1  Repeat 2  Repeat 3 | 31.97  22.83  26.35 | 38.42  37.05  27.42 | 29.97  37.60  38.94 | 19.88  32.75  17.22 | 13.68  7.50  9.45 |
|  | Average | 27.05 ± 4.61 | 34.30 ± 5.99 | 35.50 ± 4.84 | 23.28 ± 8.31 | 10.21 ± 3.16 |

Fitting curve equations:

S_p@MOF_ = S_0_ + A(1 – e^-kt^)

S_0_ = 10 nm

S_p@MOF_: Size of the protein@MOF (nm)

t: Time (s)

k: Size growth rate constant (1/s)

Ak: dS_p@MOF_ /dt (t=0) = initial rate of growth (nm/s)

Table S4. Elemental analysis for MOF and protein@MOF in the presence of different surfactants.

| Surfactant |  |  | CTAB | Triton X-100 | GMO | Lecithin |
| --- | --- | --- | --- | --- | --- | --- |
| Assembly | ZIF8 | BSA@ZIF8 | BSA@ZIF8 | BSA@ZIF8 | BSA@ZIF8 | BSA@ZIF8 |
| C | 41.7± 0.3 | 47.4± 0.4 | 46.5± 2.1 | 46.9± 0.2 | 47.7± 0.2 | 48.4± 1.7 |
| Zn | 24.4± 0.2 | 16.0± 0.15 | 16.9± 1.1 | 19.3± 1.2 | 17.3± 0.5 | 16.9± 0.7 |
| N | 23.1± 0.4 | 26.1± 1.2 | 25.1± 0.5 | 24.3± 1.2 | 24.4± 0.3 | 23.6± 0.7 |
| O | 9.6± 0.2 | 9.2± 1.0 | 9.6± 1.7 | 8.0± 0.5 | 9.2± 0.2 | 9.8± 0.7 |
| Na | 1.2± 0.1 | 0.8± 0 | 0.8± 0 | 0.9± 0.1 | 0.9± 0.1 | 0.8± 0.1 |
| S | - | 0.4± 0.1 | 0.5± 0 | 0.5± 0.1 | 0.5± 0.1 | 0.5± 0 |

Table S5. Surface area, pore width, and pore volume of ZIF-8 in comparison with protein@ZIF-8 in the presence and absence of various surfactants, derived from multipoint Brunauer–Emmett–Teller (BET) and Density Functional Theory (DFT) analysis.

| Sample Name | Surface Area (m^2^/g) | Correlation Coefficient | Pore width (Å) | Pore Volume (cc/g) | Fitting Error (%) |
| --- | --- | --- | --- | --- | --- |
| ZIF8 | 2060 | 0.99 | 17.25 | 0.79 | 6.62 |
| BSA-GMO@ZIF8 | 1517 | 0.99 | 17.25 | 0.55 | 7.03 |
| BSA-CTAB@ZIF8 | 1462 | 0.99 | 17.25 | 0.54 | 6.91 |
| BSA@ZIF8 | 1384 | 0.99 | 17.25 | 0.51 | 6.85 |
| BSA-TriotonX100@ZIF8 | 829 | 0.99 | 17.25 | 0.31 | 6.71 |
| BSA-Lec@ZIF8 | 582 | 0.99 | 17.25 | 0.22 | 6.79 |

Table S6. Char yield (%) for free BSA, MOF, and BSA@MOF in the presence of various surfactant, extracted from the thermogravimetric analysis.

|  | BSA | BSA@ZIF-8 | BSA-CTAB@ZIF8 | BSA-GMO@ZIF8 | BSA-Lecithin@ZIF8 | ZIF8 |
| --- | --- | --- | --- | --- | --- | --- |
| Char Yield (%) | 14.8 | 33.8 | 33.0 | 37.9 | 33.6 | 36.9 |

Table S7. Michaelis Menten constants calculated for Free HRP, HRP@ZIF-8, and HRP-Lec@ZIF-8

| Sample | Vmax (µM s⁻¹) | *K*_M_ (mM) | *k*_cat_ (s^-1^) |
| --- | --- | --- | --- |
| Free HRP | 71.551 | 0.926 | 795 |
| HRP-Lec@ZIF-8 | 15.497 | 1.08 | 172 |
| HRP@ZIF-8 | 0.259 | 1.041 | 3 |

References

[1]	a)T. Man, C. Xu, X.-Y. Liu, D. Li, C.-K. Tsung, H. Pei, Y. Wan, L. Li, *Nature Communications* **2022**, 13, 305; b)X. Wu, J. Ge, C. Yang, M. Hou, Z. Liu, *Chemical Communications* **2015**, 51, 13408; c)Y. Liu, S. Cui, W. Ma, Y. Wu, R. Xin, Y. Bai, Z. Chen, J. Xu, J. Ge, *Journal of the American Chemical Society* **2024**, 146, 12565.

[2]	A. Micsonai, F. Wien, L. Kernya, Y.-H. Lee, Y. Goto, M. Réfrégiers, J. Kardos, *Proceedings of the National Academy of Sciences* **2015**, 112, E3095.

[3]	L. Martínez, R. Andrade, E. G. Birgin, J. M. Martínez, *J. Comput. Chem.* **2009**, 30, 2157.

[4]	S. Jo, T. Kim, V. G. Iyer, W. Im, *J. Comput. Chem.* **2008**, 29, 1859.

[5]	a)G. J. Martyna, D. J. Tobias, M. L. Klein, *J. Chem. Phys.* **1994**, 101, 4177; b)S. E. Feller, Y. Zhang, R. W. Pastor, B. R. Brooks, *J. Chem. Phys.* **1995**, 103, 4613.

[6]	J. C. Phillips, D. J. Hardy, J. D. C. Maia, J. E. Stone, J. V. Ribeiro, R. C. Bernardi, R. Buch, G. Fiorin, J. Hénin, W. Jiang, R. McGreevy, M. C. R. Melo, B. K. Radak, R. D. Skeel, A. Singharoy, Y. Wang, B. Roux, A. Aksimentiev, Z. Luthey-Schulten, L. V. Kalé, K. Schulten, C. Chipot, E. Tajkhorshid, *J. Chem. Phys.* **2020**, 153, 044130.

[7]	a)J. Huang, S. Rauscher, G. Nawrocki, T. Ran, M. Feig, B. L. de Groot, H. Grubmüller, A. D. MacKerell, *Nat. Methods* **2017**, 14, 71; b)R. B. Best, X. Zhu, J. Shim, P. E. M. Lopes, J. Mittal, M. Feig, A. D. MacKerell, *J. Chem. Theory Comput.* **2012**, 8, 3257; c)A. D. MacKerell, Jr., M. Feig, C. L. Brooks, *J. Am. Chem. Soc.* **2004**, 126, 698; d)A. D. MacKerell, D. Bashford, M. Bellott, R. L. Dunbrack, J. D. Evanseck, M. J. Field, S. Fischer, J. Gao, H. Guo, S. Ha, D. Joseph-McCarthy, L. Kuchnir, K. Kuczera, F. T. K. Lau, C. Mattos, S. Michnick, T. Ngo, D. T. Nguyen, B. Prodhom, W. E. Reiher, B. Roux, M. Schlenkrich, J. C. Smith, R. Stote, J. Straub, M. Watanabe, J. Wiorkiewicz-Kuczera, D. Yin, M. Karplus, *J. Phys. Chem. B* **1998**, 102, 3586; e)J. B. Klauda, R. M. Venable, J. A. Freites, J. W. O'Connor, D. J. Tobias, C. Mondragon-Ramirez, I. Vorobyov, A. D. MacKerell, R. W. Pastor, *J. Phys. Chem. B* **2010**, 114, 7830.

[8]	W. Jorgensen, J. Chandrasekhar, J. Madura, R. Impey, M. Klein, *J. Chem. Phys.* **1983**, 79, 926.

[9]	T. Darden, D. York, L. Pedersen, *J. Chem. Phys.* **1993**, 98, 10089.

[10]	W. Humphrey, A. Dalke, K. Schulten, *J. Mol. Graph.* **1996**, 14, 33.

[11]	a)M. R. Housaindokht, M. R. Bozorgmehr, M. Bahrololoom, *J. Theor. Biol.* **2008**, 254, 294; b)S. Kaviani, M. Izadyar, M. Khavani, M. R. Housaindokht, *J Mol Liq* **2020**, 317, 113933.
